# Cellular Mechanisms of Circulating Tumor Cells During Breast Cancer Metastasis

**DOI:** 10.3390/ijms21145040

**Published:** 2020-07-17

**Authors:** Han-A Park, Spenser R. Brown, Yonghyun Kim

**Affiliations:** 1 Department of Human Nutrition and Hospitality Management, College of Human Environmental Sciences, The University of Alabama, P.O. Box 870311, Tuscaloosa, AL 35487, USA; 2 Department of Chemical and Biological Engineering, College of Engineering, The University of Alabama, Tuscaloosa, AL 35487, USA; sbrown104@crimson.ua.edu (S.R.B.); ykim@eng.ua.edu (Y.K.)

**Keywords:** circulating tumor cells, mitochondria, fluid shear stress, breast cancer, oxidative stress

## Abstract

Circulating tumor cells (CTCs) are cancer cells that detach from the primary site and travel in the blood stream. A higher number of CTCs increases the risk of breast cancer metastasis, and it is inversely associated with the survival rates of patients with breast cancer. Although the numbers of CTCs are generally low and the majority of CTCs die in circulation, the survival of a few CTCs can seed the development of a tumor at a secondary location. An increasing number of studies demonstrate that CTCs undergo modification in response to the dynamic biophysical environment in the blood due in part to fluid shear stress. Fluid shear stress generates reactive oxygen species (ROS), triggers redox-sensitive cell signaling, and alters the function of intracellular organelles. In particular, the mitochondrion is an important target organelle in determining the metastatic phenotype of CTCs. In healthy cells, mitochondria produce adenosine triphosphate (ATP) via oxidative phosphorylation in the electron transport chain, and during oxidative phosphorylation, they produce physiological levels of ROS. Mitochondria also govern death mechanisms such as apoptosis and mitochondrial permeability transition pore opening to, in order eliminate unwanted or damaged cells. However, in cancer cells, mitochondria are dysregulated, causing aberrant energy metabolism, redox homeostasis, and cell death pathways that may favor cancer invasiveness. In this review, we discuss the influence of fluid shear stress on CTCs with an emphasis on breast cancer pathology, then discuss alterations of cellular mechanisms that may increase the metastatic potentials of CTCs.

## 1. Breast Cancer and Circulating Tumor Cells

Breast cancer is the most common cancer in women. According to Cancer Facts & Figures 2020, published by the American Cancer Society, an estimated 279,100 new cases of invasive breast cancer and 42,690 deaths will occur in 2020 [1]. Both genetic and lifestyle factors contribute to the development of breast cancer. Mutations of tumor suppressor genes such as *BRCA1* and *BRCA2* are strongly associated with hereditary breast cancer, and abnormalities of other genes such as *CHECK2*, *ATM*, *PALB2*, *PTEN*, and *PT53* also increase risk [2,3]. Lifestyle factors such as obesity, hormone treatment, and a high-fat diet are positively correlated with breast cancer risk, whereas physical activity and a diet rich in vitamins, minerals, and phytochemicals may reduce the risk of breast cancer [4,5]. The breast cancer mortality rate was 33.2 per 100,000 in 1989, but this has declined to 19.8 since 2017 due in part to increased screening and advancements in diagnostic and therapeutic technologies [1]. Currently, the 5-year survival rate for those with non-metastatic breast cancer is 99%, whereas this declines steeply for metastatic breast cancer to just 27% [1]. Therefore, localized breast cancer is considered more manageable, and strategies to prevent metastasis are vital to reducing breast cancer mortality.

Metastatic progression is a primary cause of breast cancer-associated death [6,7]. Breast cancer cells may spread to the bone, lung, liver, and brain. However, metastatic patterns are not uniform and can vary by type of breast cancer. Especially, the distributions of estrogen receptor (ER), progesterone receptor (PR), and human epidermal growth factor receptor 2 (HER2) influence the metastatic potential of breast cancer. Therefore, elucidating receptor-mediated signaling and differential cellular outcomes is crucial to understanding the molecular mechanisms of breast cancer cell metastatic behavior. To progress to clinically detectable metastasis, cancer cells must undergo a metastasis cascade, as follows: primary tumor formation, local invasion, intravasation into blood or lymph, survival during circulation, implantation at a distant organ site, initial survival in a foreign microenvironment, and finally metastatic colonization [8,9]. Each step of the metastasis cascade acts as a biological barrier; thus, the majority of cells die before progressing to metastasis. In particular, when cancer cells detach from the primary site and enter the bloodstream as circulating tumor cells (CTCs), they are challenged with anoikis, a type of apoptosis caused by loss of attachment to the extracellular matrix. However, a few CTCs survive this challenge and, when coupled with a favorable microenvironment, develop into metastasis [10,11]. Although cutoffs can vary by type of tumor, five or more CTCs in a 7.5 mL blood sample is considered CTC positive in breast cancer [12,13]. An increasing number of studies have emphasized the significance of CTCs in mediating breast cancer metastasis. The presence of CTCs increases the risk of metastasis, and higher numbers of CTCs are inversely associated with progression-free survival and overall survival in patients with breast cancer [12,14,15]. CTCs have been suggested as a prognostic tool for monitoring metastasis or the efficacy of chemotherapy [16,17,18,19]. Studies have shown that potential diagnostic biomarkers representing stemness [20], immunogenic CTC [18], and signaling molecules that promote breast cancer metastasis [19] are found in CTCs. The mutation and expression levels of breast cancer-associated genes such as *BRCA* 1/2 are also detectable by liquid biopsy [15,21].

## 2. In Vitro Models of Circulating Tumor Cells for Studying Metastasis

Due to our current inability to observe and study metastasizing cells in vivo, it is necessary to engineer and model the cells’ dynamic environment in vitro. These models allow for the examination and analysis of the mechanobiology of the cells as they experience physiologically relevant stresses and the consequences thereof. The most significant aspect of this dynamic environment is the fluid shear stress (FSS) that CTCs experience in the blood stream. Here, cells encounter a moving, heterogeneous environment that is unfriendly to most cells, resulting in either their destruction or dormancy [22,23]. Conversely, CTCs that survive such stress become especially endowed with high metastatic potential. Physiological fluid flow is typically identified as blood, lymphoid, and interstitial flow [24], with blood and interstitial flow affecting CTCs the most. Physiological FSS ranges from 1 to 30 dyn/cm^2^, depending on the location (capillaries, veins, or arteries) [25]. As such, models for applying FSS need to achieve these levels of shear stress, while also considering the diameter of the simulated vessels. Moreover, the type of FSS induced is also relevant to the study target, as laminar shear stress and oscillatory shear stress do not produce the same effects [24,26]. 

Currently, there are several strategies for applying FSS to cancer cells, several of which are summarized in Table 1. Each of these models has been utilized to gain insight into various aspects of the mechanobiology of the CTCs, including their stemness [27], the generation of reactive oxygen species [28], drug resistance [29,30], promotion of the epithelial-mesenchymal transition (EMT) [31], fluctuation in hormone receptor expression [32], and facilitation of apoptosis [33], to name a few.

The syringe pump model is the simplest yet most robust CTC model. This device allows for the application of shear rates ranging from low (1 dyn/cm^2^) to high (>60 dyn/cm^2^), as per physiological standards [27,30]. The syringe pump is simple to use and stands on its own as a model for CTCs, but can also be incorporated into other models, such as perfusion and microfluidic devices for inducing flow. A primary objection to using the syringe pump exclusively is that it is a single-pass system that does not maintain a high residence time. For observations of the consequences of prolonged exposure to FSS, a peristaltic pump system would be more appropriate. This would allow a circulatory system for cells to experience shear stress over a wider range of exposure time [28,29,34,35]. Furthermore, the pulsatile flow resulting from the peristaltic pumps more closely mimics the blood flow generated by the cardiovascular system, so its physiological relevance is increased as compared to the previously discussed apparatus. Cone and plate viscometers calculate FSS in relation to torque and angular viscosity, as opposed to flow rate, as syringes and peristaltic pumps do. These constructs permit a larger number of cells to be exposed to FSS in a single experiment as the entire culture plate can be utilized, similarly to the rotational shakers [31,32,33]. These devices introduce a uniform application of FSS and therefore provide for more whole-population analysis, since all the cells undergo the same, proportional shear experience. Perfusion and microfluidic devices are smaller-scale FSS applications, but they allow for the introduction of greater complexity into the exposure to stress [36,37,38]. Microfluidic devices in particular can be designed with more complex tortuous flow paths for cells. The significance of this is reflected in some studies of complex extracellular matrices, as the layout of the cells’ path dictates their progression through the course [39]. Microfluidic devices have also been further developed as means of detecting CTC in patient blood samples, which has to the potential to carry clinical significance [40]. Finally, computational modeling allows the investigator to build the desired biological environment and introduce known variables and responses, in order to observe what is not actually visible on the clinical stage. Notably, the designs of the perfusion and microfluidic devices as well as the computational simulations create the opportunity for single-cell analysis, granting deeper insight into cell behaviors and the cues to which it responds. With these advances, it is conceivable to identify what makes CTCs capable of surviving the heterogeneous environment and progress through the metastatic cascade, rather than entering dormancy or being destroyed.

## 3. Oxidative Stress and Mitochondrial Dysfunction

In recent decades, increasing numbers of studies have suggested an association between of FSS-induced oxidative stress with CTC pathology. Loss of extracellular matrix in non-tumorigenic human mammary epithelial MCF10A cells is shown to increase ROS production [42]. Similarly, breast cancer cells treated with FSS increased the 2′, 7′-dichlorodihydrofluorescein (DCF) positive signal indicating accumulation of hydrogen peroxide [28,29,31,43]. FSS-induced ROS accumulation contributes to increased invasiveness of breast cancer cells by promoting migration and EMT, a process by which epithelial cells lose polarity but gain the motile and invasive characteristics of mesenchymal cells [31]. Oxidative stress enhances the transition to the HER2 negative phenotype in CTCs [44]. In addition, other non-cancer cells and extracellular vesicles found in the bloodstream also incorporate with CTC and enhance metastatic potentials. Sprouse et al. showed that the formation of clusters of non-tumor, myeloid-derived suppressor cells (MDSCs) and peripheral mononuclear cells (PMNs) enhances ROS production in CTCs and promotes ROS-mediated mitogenic signaling in breast cancer [45]. Fu et al. demonstrated a positive correlation between ROS and SMAD3, essential during cell adhesion via the mediation of transforming growth factor beta (TGF-β) signaling [46]. The authors also showed that primary tumor-derived exosomes (PTDEs) increase ROS production, and PTDEs directly communicate with CTCs, enhancing their survival and adhesion [46]. FSS-treated breast cancer cells isolated from stage III breast cancer patients showed an upregulation of antioxidant enzyme genes such as superoxide dismutase, catalase, and glutathione peroxidase [31], which potentially support cancer cell survival. Circulating tumor cells isolated from patients with leukemia showed a high expression of nuclear factor erythroid 2-related factor 2 (Nrf2), a transcription factor that binds to genes containing antioxidant response element (ARE) upon oxidative stress, is associated with drug resistance [47]. FSS-induced ROS may signal a change the intracellular antioxidant profiles of CTCs.

The mitochondrion is the central organelle that consumes oxygen to produce adenosine triphosphate (ATP) via oxidative phosphorylation. Electrons produced during metabolic processes such as the TCA cycle and fatty acid oxidation are processed by complex I, complex II, complex III, and complex IV in the electron transport chain. The electron transport creates an electrochemical gradient that forces ATP production via the F_1_Fo ATP synthase. Although the majority of oxygen is reduced to water by complex IV, 1–3% of the oxygen leaked from complex I and III is responsible for producing ROS such as superoxide [48,49]. Inefficient operation of the electron transport chain in the mitochondria increases ROS production, contributing to pathological processes. Accumulation of ROS also damages mitochondria, altering energy metabolism, mitochondrial DNA expression, and mitochondria-dependent death mechanisms. Tumorigenic breast cancer cells showed significantly high baseline ROS levels, associated with mitochondrial fragmentation and mitochondrial membrane potential loss, compared to the non-tumorigenic control [50]. Mitochondrial dysfunctions, including the fragmentation of mitochondrial DNA and the alteration of mitochondrial apoptosis, are reported for CTCs of various cancers [51,52]. Mitochondrially produced free radicals such as the superoxide anion are also greater in FSS-induced breast cancer cells [29]. Cancer cells exhibit altered metabolic characteristics with decreased reliance on mitochondrial energy metabolism, referred to as the Warburg effect (decreased ATP production via oxidative phosphorylation, but increased ATP production via glycolysis [53]). Glycolysis is an alternative source of ATP when mitochondrial function is devoted to other cellular functions such as the biosynthesis of macromolecules for proliferation; thus, high glycolytic activity supports rapid cancer cell proliferation.

## 4. Cell Signaling Pathways in Circulating Tumor Cells

ROS play dual roles regulating both the death and survival of cancer cells [54,55]. A surge of ROS potentiates breast cancer cell death during treatment with chemotherapeutics such as cisplatin and doxorubicin [56,57]. FSS can enhance the effects of ROS-generating drugs promoting the apoptosis of breast cancer cells [58], and the application of mitochondrial antioxidants reverses the pro-death effect of chemotherapy drugs [29]. However, moderate levels of ROS also contribute to cancer cell growth and survival by manipulating cell signaling pathways.

The extracellular signaling-regulated kinase (ERK)/mitogen-activated protein kinase (MAPK) pathway regulates the cell cycle and proliferation of cancer cells. ERK/MAPK is one of the most studied pathways in FSS-associated cancer pathology [28,31], and it is a highly activated pathway in triple negative breast cancer [59,60]. ERK is activated by ErbB family receptors including the epidermal growth factor receptor (EGFR/HER1) and other HERs. HER2, a key receptor responsible for over 20% of breast cancer [61], undergoes heterodimerization, and amplifies ERK signaling [62]. EGFR and HER2 are types of receptor tyrosine kinases, and phosphorylation of tyrosine residues recruits growth factor receptor-bound protein 2 (GRB2) and Son of Sevenless (SOS). SOS is a guanine nucleotide exchange factor that activates Ras, a small GTPase found in the membrane. GTP-bound activated Ras initiates the activation of Raf, a serine/threonine-specific protein kinase, and its downstream kinase cascades including mitogen-activated protein kinase kinase (MEK/MAPKK) followed by ERK. ERK phosphorylates cytoplasmic targets including ribosomal protein S6 kinase (RSK) and Bcl-2 family proteins such as Bcl-2, Bcl-xL and Mcl-1 [63]: RSK converts non-transformed epithelial cells to motile, mesenchymal carcinoma cells [64], and the application of an RSK inhibitor or siRNA blocks breast cancer cell initiation and proliferation [65,66]. Bcl-2, Bcl-xL, and Mcl-1 are pro-survival proteins that inhibit the formation of pro-death oligomers on the mitochondrial membrane. In addition to regulating cytoplasmic proteins, phosphorylated ERK translocated into the nucleus and activates transcription factors such as Elk-1, c-Jun, c-Fos, c-Myc, Mnk, and cAMP response element binding protein (CREB) which control metabolism, cell cycle, proliferation, and aggressiveness [67,68].

Ma et al. showed that MDA-MB-231 cells exposed to 5, 15, and 30 dyn/cm^2^ FSS increased the production of hydrogen peroxide and the migration of cancer cells [28]. Higher FSS increased levels of ERK1/2 phosphorylation [28]. The authors further demonstrated that the application of antioxidants such as *N*-acetyl-cysteine and propyl gallate, or MEK inhibitor U0126 reversed FSS-mediated breast cancer cell migration [28]. Ejaeidi et al. showed that CTCs derived from breast cancer patients increased protein levels of ERK, Akt, and survivin [69]. Choi et al. showed that MDA-MB-231 cells exposed to 5 dyn/cm^2^ bi-directional, oscillating shear stress increased stemness factors (e.g., Nanog, Oct4B, and Sox2) and EMT markers (e.g., cadherin, Twist, and Snail1) [31]. Breast cancer cells grown on an orbital shaker at 60 rpm, equivalent to 4.5 dyn/cm^2^, increased the production of ROS and reactive nitrogen species [31] upregulating ROS-responsive genes such as *Sod1, Cat, Nox1*, and *Gpx1* and NO-responsive genes such as *Nos1* and *Nos2* [31]. Co-treatment with FSS and a ROS scavenger, *N*-acetylcysteine, inhibited ROS-induced transcriptional change indicating an association between cancer cell redox status and FSS-mediated EMT [31]. Notably, Choi et al. showed the inhibitory effects of shear stress on ERK pathways, including decreased ERK phosphorylation [31]. c-Jun NH_2_-terminal kinase (JNK) is a downstream kinase activated by the ERK pathway. Although the ERK pathway did not lead to the activation of JNK in this study [31], Takabe et al. showed that FSS activates JNK, promoting mitochondrial ROS production [70]. The ERK pathway is an important player during FSS-induced alteration of survival, EMT, and stemness in breast cancer cells. However, ERK activation or inhibition may vary by cell type, duration, or intensity of FSS.

Phosphoinositide 3-kinase (PI3K) is a family of p85/p110 heterodimeric lipid kinases that regulates cancer cell proliferation, growth, motility, and survival via the activation of mammalian target of rapamycin (mTOR) [71]. Class IA PI3Ks contain regulatory (p85) and catalytic subunits (p110), continuing the signaling transduction activated by cell membrane receptors, including receptor tyrosine kinases and G-protein coupled receptors [72,73,74,75]. PI3K phosphorylates phosphatidylinositol 4,5 bisphosphate (PIP2) at the 3 position producing phosphatidylinositol-3,4,5-triphosphate (PIP3) in the membrane, whereas phosphatase and tension homolog (PTEN) dephosphorylates PIP3 inhibiting the PI3K/Akt pathway. PIP3 recruits phosphoinositide-dependent kinase 1 (PDK1), and PDK1 activates Akt, also called protein kinase B (PKB), via phosphorylation at the Thr 308 residue [76,77]. Akt is also phosphorylated by rapamycin complex 2 (mTORC2) at Ser 473 [78]. Activated Akt phosphorylates multiple downstream targets including tuberous sclerosis protein 2 (TSC2). TSC2 forms a heterodimer with TSC1 to act as a tumor suppressor, and TSC2 activates Rheb, a GTP-binding protein. However, Akt-mediated phosphorylation inhibits the formation of the TSC1-TSC2 complex causing the accumulation of GTP-bound Rheb. The Rheb-GTP activates mTORC1, which inhibits 4E-BP1 and activates S6K1/2, regulators of translation [77]. The targets of Akt also include glycogen synthase kinase 3β (GSK3β), NF-κB, and Bad, regulating cancer cell growth, death, and energy metabolism [79,80].

Activation of the PI3K/Akt pathways occurs in FSS-exposed cancer cells [38,81,82]. ROS generated during FSS [28,29,31,43] may signal activation of the PI3K/Akt pathway in breast cancer [83,84,85]. Application of a low level of FSS (1.8 dyn/cm^2^) to MDA-MB-231 breast cancer cells increased phosphorylation of the regulatory subunit of PI3K, p85, followed by phosphorylation of the Ser 473 site of Akt [38]. Activation of PI3K/Akt increased the protein levels of membrane type 1-matrix metalloproteinase (MT1-MMP) [38], a key player during extracellular matrix degradation [86], allowing cancer cell migration. Treatment with LY2294002, a PI3K inhibitor, MK-2206, an Akt inhibitor, or rapamycin, an mTOR inhibitor, reversed FSS-induced MT1-MMP upregulation [38]. FSS exposed colorectal cancer cells showed Akt-mediated upregulation of atonal bHLH transcription factor 8 (ATOH8) and yes-associated protein 1 (YAP1), transcription factors that enhance cancer cell survival, growth, metastasis, and metabolic remodeling [81,82]. In addition to FSS-induced ROS, changes in the microenvironment also contribute to ROS generation. Cancer cells are hypoxic; thus, oxygenation occurring during their entrance into the bloodstream increases the production of ROS. MCF7 breast cancer cells subjected to hypoxia (1% oxygen) followed by reoxygenation (10% oxygen) increased phosphorylation of Akt and Erk1/2 [87]. ROS regulates the PI3K/Akt pathways, but PI3K/Akt also regulates redox status in breast cancer cells [88]. Akt enhances glutathione biosynthesis via Nrf2-mediated upregulation of glutathione synthase and glutathione reductase [88].

## 5. Alteration of Mitochondrial Death Mechanism

Alteration of apoptotic death including impaired pro-apoptotic or excess anti-apoptotic mechanisms is commonly found in cancer cells. Under normal physiological conditions, unwanted or damaged cells undergo apoptotic death. Binding of a ligand to a death receptor such as the Fas receptor and tumor necrosis factor-α (TNF-α) receptor initiates extrinsic apoptosis activating an initiator caspase followed by activator caspases, or integrates with mitochondria-mediated intrinsic apoptosis. Signaling proteins activated by an extrinsic pathway such as Bid, or accumulation of intracellular stimuli such as ROS and calcium activate the pro-apoptotic Bcl-2 proteins, Bax and Bak. These pro-death proteins are oligomerized on the mitochondrial membrane, causing the mitochondrial membrane to become permeable. Cytochrome c is released from mitochondria upon loss of mitochondrial membrane integrity, and forms the apoptosome, a protein complex with an apoptotic protease activating factor 1 (Apaf-1), activating caspase 9 and downstream activator caspases including caspase 3 [89,90].

The application of gene set enrichment analysis showed that CTC derived from breast cancer patients decreased apoptosis [91,92], but increased the pathways involved in metastasis, cholesterol biosynthesis, and angiogenesis [91]. Human breast cancer cells MDA-MB-231, MCF7, and ZR75-1 cultured in suspension, mimicking the bloodstream environment, lowered protein levels of Fas, TNF-α receptor, and death receptor 5 (DR5), death receptors that initiate extrinsic apoptosis [93], and this impaired the activation of caspase 3 compared to the adherent group [93]. Consistently, the application of low shear stress (2 dyn/cm^2^) to MDA-MB-231 cells blocks Fas-induced extrinsic apoptosis [52]. FSS exposure impaired the conversion of pro- to the active form of caspase-8 preventing truncation of Bid. This impairment led to the failure of downstream executor caspase activation [52]. However, the inhibitory effects of FSS on apoptosis may vary by differential receptor profiles the breast cancer cells. Fu et al. showed that triple negative MDA-MB-231 breast cancer cells were more resistant against FSS-mediated oxidative stress and caspase 3/7 activation than hormonal receptor-expressing MCF7 cells [29].

Previous clinical research using CTCs isolated from patients with metastatic breast cancer suggested B-cell lymphoma 2 (Bcl-2) as a potential tool for assessing therapeutic efficacy [94,95]. Bcl-2 is an anti-apoptotic member of the Bcl-2 family of proteins along with Bcl-xL and Mcl-1. These anti-apoptotic Bcl-2 proteins are expressed on the mitochondrial membrane and bind directly to pro-death Bax and Bak, thus preventing apoptotic cell death. Thangavel et al. showed upregulation of the Bcl-2 protein in CTC collected from mice transplanted with triple negative human breast cancer [91]. ROS upregulate the mitochondrial anti-apoptotic proteins including Bcl-xL and Bcl-2 via activation of the transcription factors NF-κB, Nrf-2, and HIF-1α [96,97,98]. ROS also manipulate expression of the pro-apoptotic proteins, including Bad and Bim, via the ERK/MAPK and PI3K/Akt pathways [99,100,101]. Thus, FSS-induced ROS may change the proportions of pro- and anti-apoptotic gene expression, altering the apoptotic pathway. Although it is still unclear whether high or low levels of anti-apoptotic Bcl-2 proteins in CTCs is correlated with breast cancer metastasis in humans [94,95], mice intravenously injected with MDA-MB-435 breast cancer cells overexpressing Bcl-xL increased formation of secondary tumors [102]. CTCs expressing Bcl-2 were resistant to anoikis [103]. This study also showed that higher Bcl-2 expression on CTCs is correlated with higher levels of adhesion molecules including E-selectin, ICAM-1, and VCAM-1 [103]. Strategies enhancing apoptosis such as caspase 3 activation and Bcl-xL depletion are correlated with a decreased number of CTCs and metastatic colonization [104].

Previous clinical research using CTCs isolated from patients with metastatic breast cancer suggested B-cell lymphoma 2 (Bcl-2) as a potential tool for assessing therapeutic efficacy [94,95]. Bcl-2 is an anti-apoptotic member of the Bcl-2 family of proteins along with Bcl-xL and Mcl-1. These anti-apoptotic Bcl-2 proteins are expressed on the mitochondrial membrane and bind directly to pro-death Bax and Bak, thus preventing apoptotic cell death. Thangavel et al. showed upregulation of the Bcl-2 protein in CTC collected from mice transplanted with triple negative human breast cancer [91]. ROS upregulate the mitochondrial anti-apoptotic proteins including Bcl-xL and Bcl-2 via activation of the transcription factors NF-κB, Nrf-2, and HIF-1α [96,97,98]. ROS also manipulate expression of the pro-apoptotic proteins, including Bad and Bim, via the ERK/MAPK and PI3K/Akt pathways [99,100,101]. Thus, FSS-induced ROS may change the proportions of pro- and anti-apoptotic gene expression, altering the apoptotic pathway. Although it is still unclear whether high or low levels of anti-apoptotic Bcl-2 proteins in CTCs is correlated with breast cancer metastasis in humans [94,95], mice intravenously injected with MDA-MB-435 breast cancer cells overexpressing Bcl-xL increased formation of secondary tumors [102]. CTCs expressing Bcl-2 were resistant to anoikis [103]. This study also showed that higher Bcl-2 expression on CTCs is correlated with higher levels of adhesion molecules including E-selectin, ICAM-1, and VCAM-1 [103]. Strategies enhancing apoptosis such as caspase 3 activation and Bcl-xL depletion are correlated with a decreased number of CTCs and metastatic colonization [104].

## 6. Alteration of Mitochondrial Energy Metabolism

Metabolic remodeling such as decreased oxidative phosphorylation in mitochondria and increased glycolysis in the cytoplasm (Warburg effect), is a hallmark of cancer [53,105,106,107]. Breast cancer cells can compensate energy deficits during impaired oxidative phosphorylation by upregulating genes involved in glycolysis [108]. Glycolysis provides ATP more rapidly than oxidative phosphorylation, and high glycolytic activity supports rapid cancer cell proliferation. Indeed, invasive cells show higher glycolysis than less invasive cancer cells [109,110]. While the Warburg effect is an important metabolic phenotype in cancer, cancer cells are also known to have high metabolic flexibility in the presence of shear stress. FSS can shift energy metabolism to glycolysis via the upregulation of hexokinase in colorectal cancer [82]. In addition to glucose metabolism, metastatic breast cancer cells show a higher dependency on fatty acid oxidation [111]. FSS has been previously reported to alter mitochondrial bioenergetic profiles in vascular endothelium [112]. Recently, Huang et al. demonstrated that the application of a physiological range of FSS (5–20 dyn/cm^2^) is capable of shifting cancer cell energy metabolism to glycolysis in colorectal cancer models. This study suggested that FSS-induced Akt signaling increases the expression of ATOH8, and the upregulation of ATOH8 ultimately promotes glycolysis by activating hexokinase transcription [82]. Similarly, Chen et al. showed that advanced tumor states and EMT phenotypes are associated with increased expression of phosphoglycerate kinase 1 and glucose-6-phosphate dehydrogenase in CTCs, enzymes that conduct cytoplasmic glycolysis and the pentose phosphate pathway, respectively [113].

F_1_Fo ATP synthase, an enzyme complex located in the mitochondrial inner membrane, plays a central role in cellular energy metabolism. F_1_Fo ATP synthase has multiple subunits, including the F_1_ complex with a, b, c, α, β, γ, δ and ε subunits and the oligomycin sensitivity-conferring protein (OSCP). The Fo complex has a, b, and c subunits. Studies have shown an association between breast cancer and mutations on *ATP6* and *ATP8* genes that encode the a-subunit of the F_1_Fo ATP synthase [114,115,116]. The a-subunit is critical in the rotation of F_1_Fo ATP synthase, a necessary process during ATP production; therefore, *ATP6* and *ATP8* mutations negatively influence mitochondrial energy metabolism [117]. In addition to mutation, breast cancer pathology alters the abundance of other subunits of the F_1_Fo ATP synthase [118]. Tissue biopsy samples obtained from patients with breast, gastric, and esophageal cancer showed depletion of the β-subunit of the F_1_Fo ATP synthase [118]. The β-subunit interacts with ADP and releases ATP, and it also binds to various proteins that regulate the efficiency of mitochondrial energy metabolism [119,120]. The F_1_Fo ATP synthase inhibitor (IF1) is a protein that targets the α and β subunits of F_1_Fo ATP synthase, blocking ATP production [121]. The upregulation of IF1 in human breast cancer cells is associated with impaired mitochondrial energy metabolism, enhanced glycolysis, lactate production, and fatty acid oxidation [122]. Analysis with CTCs isolated from breast cancer patients showed downregulation of acetyl-CoA carboxylase, a key enzyme during fatty acid synthesis [91] suggesting the alteration of fat metabolism also occurs in CTCs. The c-subunit ring of F_1_Fo ATP synthase exhibits a large non-selective mitochondrial channel activity hindering mitochondrial energy metabolism [120,123,124,125], and inhibition or mutation of the c-subunit prevents cell death associated with oxidative stress [123]. Interestingly, F_1_Fo ATP synthase is also found on the surface (ecto) of human breast cancer cells, MDA-MB-231 [126]. The ecto F_1_Fo ATP synthase acts as an ApoA1 receptor and regulates cellular uptake of lipoproteins and angiogenesis [127]. The application of angiostatin, an inhibitor of cell migration and the proliferation, binds to the α-subunit of the ecto F_1_Fo ATP synthase and inhibits angiogenesis [128]. The expression of the β-subunit of the ecto F_1_Fo ATP synthase is also higher in breast cancer cells than normal cells [129], and application of an aptamer or an antibody targeting the β-subunit induced cytotoxicity in various epithelial cells including breast cancer cells [129,130]. Inhibition of the β-subunit increased apoptosis and blocked phosphorylation of ERK and Akt, thus the ecto β-subunit may regulate survival genes that are under the control of the ERK and Akt pathways [130].

## 7. Antioxidant and Breast Cancer Metastasis

Due to the roles of ROS in manipulating the signaling pathways, energy metabolism and death mechanisms of breast cancer cells, treatments with antioxidants potentially exhibit therapeutic effects against CTC-associated metastasis [131]. Tam et al. injected luciferase labeled-human breast cancer cells, MDA-MB-231, into the mammary fat pads of immunodeficient mice to develop primary tumors. The animals were then fed with a normal vs. an antioxidant-rich diet, including vitamin E (99 IU/kg) and pterostilbene (40 μg/kg) [131]. The mice in the experimental group demonstrated a significantly reduced number of CTCs, and these dietary antioxidants also reduced the size of the primary tumor [131]. Vitamin Es including both tocopherols and tocotrienols are well-studied antioxidants. Vitamin E scavenges singlet oxygen [132] and prevents mitochondrial dysfunction associated with the accumulation of superoxide [133,134,135]. Therefore, vitamin E may be beneficial in preventing the mitochondrial remodeling that occurs during an FSS-induced ROS challenge. Treatment with vitamin E attenuated estrogen-induced nitrosative and oxidative stress in both in vivo and in vitro breast cancer models, while it enhanced expression of antioxidant enzymes such as superoxide dismutase, glutathione peroxidase, and catalase via upregulation of Nrf2 [136,137]. Vitamin E manipulates redox-sensitive cell signaling pathways: Vitamin E decreases phosphorylation of ERK and Akt in breast cancer cells [131,138,139] changing the activities of downstream targets that regulate glycolysis, apoptosis, and cell cycles [131,138,140,141]. In addition to its traditional antioxidant functions, vitamin E also regulates mitochondrial genes and exhibits anticancer effects. The application of mitochondrially targeted vitamin E lowered mitochondrial DNA transcription in mouse model of HER2^high^ breast cancer [142]. Vitamin E may bind directly to Bcl-2 family proteins regulating their activities or mitochondrial permeability transition [133,143]. Mitochondrial vitamin E increases the loss of mitochondrial membrane potential and the production of ROS potentiating breast cancer cell death [142]. Vitamin E prevents the production of inflammatory cytokines that are causative for breast cancer development [144].

Vitamin C donates an electron, to impart antioxidant properties and the ability to neutralize free radicals. Low levels of plasma vitamin C and high levels of lipid peroxidation are associated with metastatic breast cancer [145], while increased dietary vitamin C is associated with a decreased risk of breast cancer related mortality [146]. In addition to its well-studied antioxidant function, vitamin C can also act as a pro-oxidant and cause cytotoxicity in cancer cells [147]. Supratherapeutic concentrations of vitamin C decrease the viability of both non-metastatic and metastatic breast cancer cells and support chemotherapy treatment [148,149,150]. Loss of extracellular matrix adhesion in primary cancer cells leads to intravasation, releasing CTCs. Studies suggested potential roles of vitamin C in preventing breast cancer metastasis via the regulation of the extracellular matrix [151,152]. An electron from vitamin C can be used to reduce ferric iron to ferrous iron, and this supports the action of lysyl hydroxylase and prolyl hydroxylase, iron binding enzymes required during collagen synthesis. Transgenic mice lacking gulonolactone oxidase, a key enzyme for vitamin C synthesis, showed a poorly defined collagenous barrier, whereas vitamin C supplemented mice showed optimal extracellular matrix formation [151,153]. Treatment with a high (millimolar) concentration of vitamin C increased protein levels of E-cadherin, a key protein for cell-extracellular matrix adhesion in the BCap-37 human breast cancer cell [152]. Micromolar levels of vitamin C impaired the assembly of the actin filament via downregulation of YAP1, and this decreased motility of MDA-MB-231 cells [154]. YAP1 is a transcriptional co-activator that regulates angiogenesis, apoptosis, DNA repair, and energy metabolism [155]. FSS is shown to increase the nuclear translocation of YAP1 in epithelial cells [156].

Carotenoids, pigments found in fruits, vegetables, and algae, play a role in redox homeostasis. Examples include β-carotene, lycopene, lutein, astaxanthin, and fucoxanthin. Some carotenoids such as β-carotene are converted into vitamin A. Vitamin A regulates genes that control proliferation and differentiation by interacting with nuclear receptors such as the retinoic acid receptor (RAR) and retinoid X receptor (RXR). Clinical studies with breast cancer patients showed an association between stage III metastatic cancer, metabolic alteration, and decreased serum concentrations of β-carotene and vitamin A [157,158]. β-carotene regulates the expression of oxidative stress-sensitive genes via Nrf2 [159]. β-carotene also enhances the apoptosis of breast cancer cells by enhancing the activation of caspase 3 [159]. A combination of vitamin A with a chemotherapy drug or pro-apoptotic molecules potentiates breast cancer cell death by decreasing pro-survival Bcl-2, increasing pro-death Bax, and activating caspases [160,161,162]. CTCs isolated from breast cancer patients demonstrate an altered apoptotic pathway [91,92], and FSS exposed breast cancer cells decrease the abundance of pro-apoptotic proteins such as caspase 3 [29,52]. Therefore, treatment with β-carotene or vitamin A may prevent FSS-mediated resistance against apoptotic death. Treatment with fucoxanthin showed concentration-dependent suppression of PI3K/Akt phosphorylation and NF-κB protein levels in human breast cancer cells [163]. The activation of PI3K/Akt contributes to metastatic phenotypes of breast cancer. In particular, FSS increases the phosphorylation of PI3K and Akt, increasing the motility of breast cancer [38]. NF-κB is a proinflammatory transcription factor that promotes the development of invasive breast cancer. NF-κB has previously been shown to bind to the promoter region of Bcl-xL and Bcl-2, resulting in a pro-survival response [164,165]. Therefore, treatment with fucoxanthin may help to decrease the malignant phenotypes of CTCs and prevent the metastasis of breast cancer. Despite the anti-metastatic properties of antioxidants, opposing effects of antioxidants have also been documented. Zhen et al. show that treatment with *N*-acetyl cysteine protects CTCs from oxidative stress-associated damage and promotes the survival of breast cancer cells [166]. Studies also suggest that supplementation with antioxidants including vitamin E, C and carotenoids may interfere with the potency of chemotherapy drugs and enhance the recurrence of breast cancer [167,168,169]. Due to these mixed results, further investigation into specific conditions like concentration and duration of antioxidant treatment, severity of cancer, and cancer cell characteristics may be useful to determine the exact therapeutic role of antioxidants in CTC-mediated metastasis.

## 8. Conclusions

Breast cancer cells traveling in the bloodstream undergo various modifications. FSS occurs in a dynamic circulation environment that can challenge cancer cells; however, it can also activate survival mechanisms that potentially increase the risk of metastasis. In this review, cellular mechanisms that may explain CTC-induced metastasis in breast cancer were discussed, including of redox homeostasis, cell signaling pathways, energy metabolism, and death mechanisms (Table 2). The effect of mitochondria on metastasis via their role in generating ROS, performing oxidative phosphorylation, and governing death mechanisms like apoptosis and mitochondrial permeability transition pore opening was highlighted. Mitochondrial dysfunction that occurs in cancer cells during circulation may influence ROS-sensitive cell signaling, energy remodeling, growth, and survival of these cancer cells. Therefore, further investigations that elucidate the cellular and molecular mechanisms by which CTCs gain the metastatic phenotype and new strategies that inhibit CTC modification will be important in preventing breast cancer metastasis.

## Figures and Tables

**Table 1:** Methods for applying fluid shear stress to cancer cells.

Flow Apparatus	Application	References
Syringe pump	Single-pass expulsion of cells from syringe through attached tubing into collection tube	[27,30]
Peristaltic pump	Circulatory system that permits multiple passes of cells through a closed loop, permitting the application of wall shear stress and laminar shear stress	[28,29,34,35]
Cone and plate viscometer	Stationary plate positioned beneath a rotating cone in a circulating water bath, permitting a uniform shear rate applied to the cell suspension	[31,33]
Orbital/rotary shaker	Cells in culture containers placed on rotating shakers at a programmed speed (rpm), permitting continuous exposure to fluid shear stress	[31,32]
Microfluidic devices	Polymeric devices with inlet and outlet ports that permit the flow of cells through designed channels, ranging in complexity, permitting the observation of cellular behavior	[36]
Parallel plate perfusion device	Stationary device with a polymeric distributor, a silicon gasket and a glass coverslip; the distributor contains the inlet and outlet ports, as well as the vacuum slot	[37,38]
Computational modeling system	Simulation of metastasizing cells in a 3D environment	[41]

**Table 2:** Current research reporting cellular the mechanisms of the FSS-associated alteration of breast cancer cells applying in vitro and in vivo models.

Mechanisms	References
Oxidative stress and antioxidant	[28,29,31,43,50,131]
Cell Signaling	
MAPK/ERK	[28,31,131]
PI3K/Akt	[38,86,131]
JNK	[70]
RANK	[19]
Apoptotic Pathway	[29,52,91,93,94,95,102,103,104]
Energy Metabolism	[170]
EMT	[20,21,27,31,104,170,171]
Stemness	[31]
Morphology	[171,172]
